# Divergent Cortical Generators of MEG and EEG during Human Sleep Spindles Suggested by Distributed Source Modeling

**DOI:** 10.1371/journal.pone.0011454

**Published:** 2010-07-07

**Authors:** Nima Dehghani, Sydney S. Cash, Chih C. Chen, Donald J. Hagler, Mingxiong Huang, Anders M. Dale, Eric Halgren

**Affiliations:** 1 Department of Radiology, University of California San Diego, San Diego, California, United States of America; 2 Martinos Center for Biomedical Imaging, Harvard Medical School, Boston, Massachusetts, United States of America; 3 Laboratory for Computational Neuroscience, Unité de Neurosciences, Information et Complexité (UNIC), CNRS, Gif-sur-Yvette, France; 4 Department of Neurology, Massachusetts General Hospital, Boston, Massachusetts, United States of America; 5 Department of Neurology, National Taiwan University Hospital, Taipei, Taiwan; 6 Department of Neurosciences, University of California San Diego, San Diego, California, United States of America; Cuban Neuroscience Center, Cuba

## Abstract

**Background:**

Sleep spindles are ∼1-second bursts of 10–15 Hz activity, occurring during normal stage 2 sleep. In animals, sleep spindles can be synchronous across multiple cortical and thalamic locations, suggesting a distributed stable phase-locked generating system. The high synchrony of spindles across scalp EEG sites suggests that this may also be true in humans. However, prior MEG studies suggest multiple and varying generators.

**Methodology/Principal Findings:**

We recorded 306 channels of MEG simultaneously with 60 channels of EEG during naturally occurring spindles of stage 2 sleep in 7 healthy subjects. High-resolution structural MRI was obtained in each subject, to define the shells for a boundary element forward solution and to reconstruct the cortex providing the solution space for a noise-normalized minimum norm source estimation procedure. Integrated across the entire duration of all spindles, sources estimated from EEG and MEG are similar, diffuse and widespread, including all lobes from both hemispheres. However, the locations, phase and amplitude of sources simultaneously estimated from MEG versus EEG are highly distinct during the same spindles. Specifically, the sources estimated from EEG are highly synchronous across the cortex, whereas those from MEG rapidly shift in phase, hemisphere, and the location within the hemisphere.

**Conclusions/Significance:**

The heterogeneity of MEG sources implies that multiple generators are active during human sleep spindles. If the source modeling is correct, then EEG spindles are generated by a different, diffusely synchronous system. Animal studies have identified two thalamo-cortical systems, core and matrix, that produce focal or diffuse activation and thus could underlie MEG and EEG spindles, respectively. Alternatively, EEG spindles could reflect overlap at the sensors of the same sources as are seen from the MEG. Although our results generally match human intracranial recordings, additional improvements are possible and simultaneous intra- and extra-cranial measures are needed to test their accuracy.

## Introduction

Among the most prominent oscillations in the human EEG are sleep spindles, repeated bursts of 10–15 Hz waves waxing and waning over about a second, mainly in stage 2 NREM sleep [1], [2]. Spindles also occur in lower mammals [3], where they have been intensively studied during sleep, barbiturate anesthesia, and in vitro, as a prototype of thalamocortical synchronization [4], [5], [6], [7], with a possible role in memory consolidation and regulation of arousal [8], [9]. More generally, spindles may represent a basic thalamo-cortical mechanism for modulating widespread cortical areas and synchronizing their interactions [8].

In animals, direct thalamic and cortical recordings have found multiple asynchronous spindle generators in some preparations whereas others find a widespread synchrony [4], [6], [7]. Most studies showing asynchrony were conducted under anesthesia, or *in vitro* [6], [9], [10]. Contreras [6] showed experimentally that the synchrony of thalamic spindles depended upon cortico-thalamic projections, and proposed mechanisms that have been replicated in computational models [11], [12].These models are based on intracellular studies demonstrating that spindles emerge from interactions between inhibitory cells in the thalamic reticular nucleus and bursting thalamocortical neurons, that entrain this rhythm on the connected cortical areas [13].

In humans, the high correlation of spindle discharges across widely dispersed scalp EEG channels has been taken to imply a widespread synchrony of spindle generators across the cortical mantle [6]. However, EEG spindles often have lower frequencies over frontal as compared to parietal leads [2], especially toward the end of the spindle burst [14], suggesting that at least two spindle generators may be active. Indeed, a variety of source estimation techniques have found that four sources, placed in the deep parieto-central and fronto-central regions bilaterally, are adequate to explain most of the variation in spindles, including the tendency for frontal spindles to be slower [15], [16], [17], although Gumenyuk et al [18] estimated that the sources for faster and slower spindle components as overlapping. Conversely, Shih et al. [19] found that more sources were needed to model their measurements but this could be related to their subjects being sedated [6]. Further evidence against a monolithic distributed synchronous generator has been found in comparisons of simultaneous EEG and MEG (magnetoencephalogram) recordings during spindles. Spindles may appear only in the MEG, only EEG, or in both modalities [15], [17], [20], [21], [22]

We re-examined these issues in a recent study [23], using high density EEG and MEG. While we replicated the previous findings that EEG signals during spindles are highly coherent across the entire scalp, simultaneous MEG signals were generally incoherent with each other and with the EEG. Further, we showed that many spindles occurring in multiple MEG channels are not readily apparent in the simultaneous EEG [Dehghani et al, submitted]. These findings seem to contradict the well-known fact that MEG and EEG reflect the same cortical generating dipoles, although the biophysics of their projection to their respective sensors are somewhat different. Thus, one might expect that if their sources were separately estimated and then compared, they would be found to be more similar than the MEG and EEG signals at their sensors. That is, projecting the EEG and MEG signals back to their sources might remove, at least in part, differences in their manifestations that are due to their divergent projections from sources to sensors.

In order to evaluate the possibility that some of the differences between MEG and EEG spindles noted at the sensors would be attenuated at their cortical sources, we performed source localization on simultaneously recorded MEG and EEG. A distributed cortically-constrained noise-normalized minimum norm inverse solution was applied to individual spindles, and the time-courses and spatial patterns were compared between solutions based on EEG and those based on MEG. Inverse estimates based on both modalities combined were also calculated. We find that, at the source level, EEG and MEG remain poorly correlated and with divergent characteristics. Sources derived from EEG are widespread, synchronous, and consistent across time and spindles, whereas those derived from MEG gradiometer recordings are relatively focal, independent and variable. We hypothesize that MEG versus EEG may be differentially sensitive to different thalamocortical systems engaged in spindle generation.

## Methods

### Ethics Statement

These studies were approved by the Institutional Review Boards of the University of California at San Diego and Massachusetts General Hospital, and were performed after written informed consent in conformity with the principles expressed in the Declaration of Helsinki.

### Participants and Recordings

**Figure 1:**
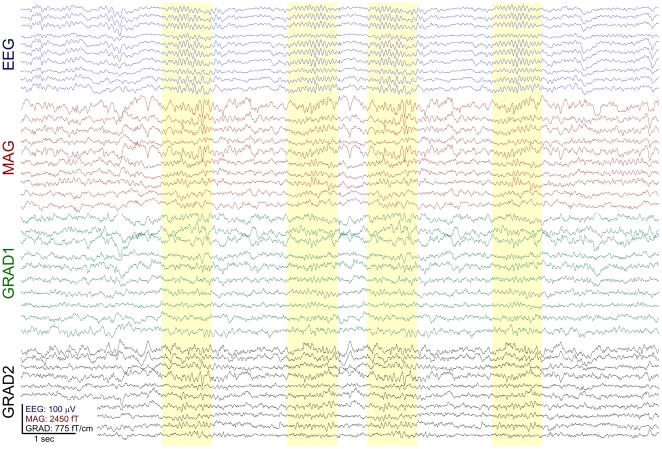
Example spindles. Selected spindles in sample EEG and MEG channels are highlighted in yellow. Complete recording profiles are shown in Figure S1.

We recorded the electromagnetic field of the brain during sleep from seven healthy adults (3 males, 4 females, ages 20–35). Participants denied neurological problems including sleep disorders, epilepsy, or substance dependence, were taking no medications, and did not consume caffeine or alcohol on the day of the recording. We used a whole-head MEG scanner (Neuromag Elekta) within a magnetically shielded room (IMEDCO, Hagendorf, Switzerland) and recorded simultaneously with 60 channels of EEG and 306 MEG channels. MEG SQUID (super conducting quantum interference device) sensors are arranged as triplets at 102 locations; each location contains one “magnetometer” and two orthogonal planar “gradiometers” (GRAD1, GRAD2). Locations of the EEG electrodes on the scalp of individual subjects were recorded using a 3D digitizer (Polhemus FastTrack). HPI (head position index) coils were used to measure the spatial arrangement of head relative to the scanner. Four subjects had a full night's sleep in the scanner, and three had a daytime sleep recording (2 hours). Sampling rate was either 1000 Hz (down sampled by factor of 2 for the final analysis) or 600 Hz. The continuous data were low-pass filtered at 40 Hz. An independent component analysis (ICA) algorithm was used to remove ECG contamination [24]. Stage 2 sleep and spontaneous spindles were identified using standard criteria by three electroencephalographers (please see Figure 1 for representative channels and Figure S1 for all channels) [25].

### Anatomical MRI and Cortical Reconstruction

Anatomical MRI images were acquired on 1.5 Tesla scanners using an MPRAGE (Magnetization Prepared Rapid Gradient Echo) sequence (on Siemens scanners) or its equivalent on a GE scanner. These T1 images were segmented using Freesurfer [26] and the tessellated border between white matter and gray matter was chosen as the representative cortical surface for forward/inverse solutions [27]. The tessellated surface of each hemisphere had ∼140,000 vertices. For computational efficiency, each hemisphere's surface was decimated down to ∼3200 dipole seeding points. This decimation provides ∼7 mm spacing between seeded dipoles across the cortical surface. For better visualization, tessellated surfaces were inflated to unfold cortical sulci [28]. Cortical parcellation was performed to create a “mid-brain mask” in order to exclude non-cortical structures (such as basal ganglia and corpus callosum) from inverse solution results as these structures are not likely to generate significant MEG signal [29].

### Source localization

Realistically shaped models have higher prediction accuracy for source localization in comparison to spherical shell models [30], [31], [32], [33]. In our source localization methods, we used a three-shell realistically shaped boundary element head model (BEM) constructed from tessellated surfaces of inner-skull, outer-skull and outer-skin (scalp) [33]. It has been suggested that single shell BEM has adequate accuracy for forward-inverse calculation of MEG but not for EEG recordings [32], [34]. However, as we needed to have an unbiased comparison of MEG and EEG source localization, we used the three shell BEM model for both. A forward BEM transformation matrix was calculated based on the spatial configuration of EEG electrodes, information from a three-shell boundary element model (BEM) and the location of dipole seeds on the reconstructed surface.

Dynamic statistical parametric mapping (dSPM) was used to estimate the cortical generators of measured signal at EEG or MEG sensors, as described by Dale and colleagues [35]. This inverse solution is a minimum norm procedure [36], where the source dipoles are constrained to lie in the reconstructed cortical surfaces [27], and the estimate is normalized for noise sensitivity so that statistical significance rather than dipole moment is mapped on the cortical surface. This results in a relatively uniform point-spread function between different dipole locations [37].

Since in the current study, no *a priori* assumptions were made about the local dipole orientation, three components were required for each location. A sensitivity-normalized estimate of the local current dipole power (sum of squared dipole component strengths) at each source location was calculated [27], [38]. Spindle waveforms at the sources were tested for the null hypothesis that the signal was noise. The noise covariance was calculated in one of two ways. In one method, 100 epochs, each 600 ms long, were chosen for each patient. Although occurring in the temporal vicinity of the sleep spindles, these epochs were chosen because they lacked spindle discharges or other sleep grapho-elements. These epochs were filtered with the same filter as was used for the spindle recordings, averaged together, and then used for noise covariance calculations in the same way as the baseline pre-stimulus period is used when dSPM is applied to event-related potentials [35]. As is shown below, similar results were obtained using noise covariance estimates derived from empty room measurements. The significance of response at each site was calculated using an F-test [35], [39]. The resultant dynamic statistical parametric maps (dSPM) were visualized on individual's inflated cortical surfaces [35]. Group averages were made by aligning the sulcal-gyral patterns of individual subjects and minimizing stretching of the surface while morphing into a reference sphere [40]. This approach provides statistical parametric maps of cortical activity, similar to the statistical maps typically generated using fMRI, or PET data, but with a temporal resolution limited by the 500/600 Hz sampling rate. Sources were not estimated for surfaces that represented deep white matter, ventricles, or noncortical structures unlikely to generate extracranial MEG or EEG signals.

For each spindle, the maximum ECD strength of a given dipole was calculated. The average of these maximums within each subject were mapped on that subject's reconstructed cortical surface for visual comparison. These methods were repeated for dSPM calculated from EEG alone (‘EEG-dSPM’), MEG alone (‘MEG-dSPM’), or both simultaneously (‘MEG+EEG-dSPM’).

### Cross correlation and coherence of source space solutions

For a given spindle, “within-modality” correlations of EEG-dSPM solutions were measured by calculating the cross correlations of activity of all possible pairs of dipoles during spindling. Self-pairs were excluded. Averaging of these cross correlations aross spindles and then across subjects yielded the net “within modality” cross correlation of EEG-dSPM. The “within modality cross correlation of MEG-dSPM” was calculated in an analogous fashion.

For a given spindle, the “between modality” correlation was measured by finding the cross-correlations of activity of a given dipole as estimated from EEG with dSPM, with the activity of the same dipole as estimated from MEG. These “between modality” cross-correlations were averaged across dipoles, spindles and subjects. Analogous measures were obtained for coherence.

When differential coherence in nearby frequency bands needed to be estimated, Capon's nonparametric spectral estimation which is known as the “minimum variance distortionless response” (MVDR) was used. MVDR spectral estimation is based on the output of a bank of filters where the bandpass filters are data and frequency dependent [41]. The MVDR may be advantageous over Welch's method in distinguishing the coherences of nearby frequencies. The fact that cross correlation of EEG-dSPM and MEG-dSPM were very low (see below) shows that these solution time courses do not have linear dependence.

## Results

Based on standard clinical criteria, we used the EEG to select 85 spindles occurring in stage 2 sleep from the 7 subjects (∼12 from each subject). Spindles immediately preceded by Vertex-waves or K-complexes were not chosen. Spindle duration mean and std were 721±235 ms (range 483 to 1123 ms). Spindle synchrony was examined between cortical locations in source space, using activity time-courses inferred from a distributed inverse solution.

### Effects of different noise normalization procedures

**Figure 2:**
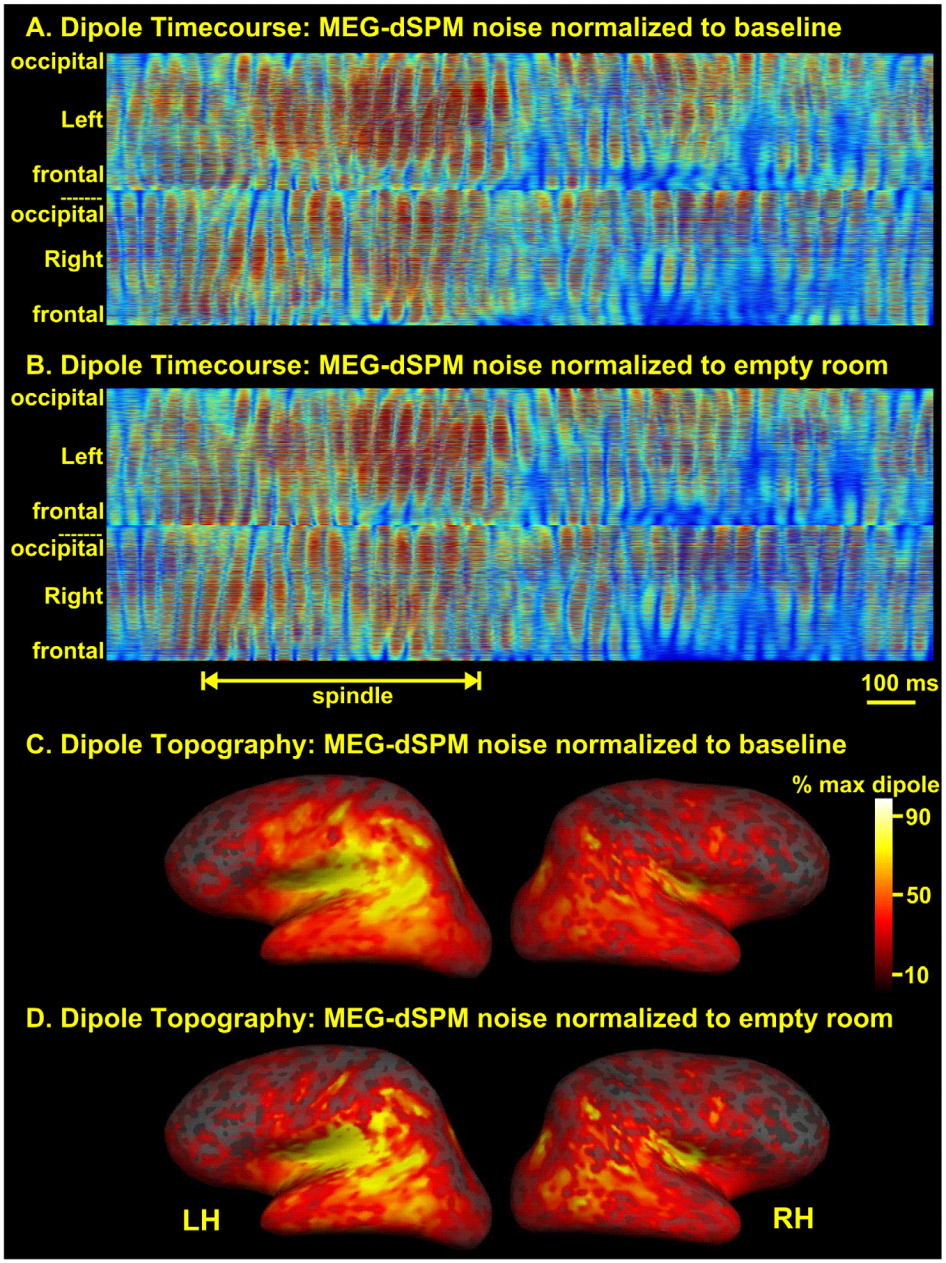
Comparison of source localization using different noise estimates. A. Spindle MEG-dSPM normalized with noise covariance calculated from averages of sleep epochs lacking grapho-elements. Normalized dipole strength for each of 6500 cortical dipoles is plotted as a horizontal line; red is high activity. B. MEG-dSPM from the same spindle, but normalized with noise estimates from empty room recordings. C, D. The activity shown in panels A and B were averaged over the course of the spindle and plotted on the reconstructed cortical surface of this subject. Very similar cortical activity patterns were inferred using the baseline (C) as compared to the empty room (D) noise covariance calculations.

For evoked responses, noise covariance in the dSPM procedure is calculated from either averaged or un-averaged pre-stimulus baseline activity [35]. Since there was no stimulus in the current study, noise covariance was calculated from ∼100 epochs (with a duration of 600 ms each) selected from stage two sleep recordings which had been band-passed at the spindle frequency, i.e. 10–15 Hz. Epochs were chosen which lacked any recognizable graphoelements or oscillatory features that could be categorized as one of the signatures of sleep. These epochs were averaged and the diagonal elements of the second power of the standard deviation of the “sample*channel” matrix was used as the noise covariance matrix [27], [35], [37]. Since electromagnetic activity was not time-locked in any way with the onset of these epochs, the averaging procedure tended to represent the sensor distribution of the biological, instrumentation and environmental noise. In four subjects, an empty room recording provided an estimate of the instrumentation and environmental noise. Inverse solutions using the covariance matrix from this recording provided very similar results to those obtained using the ‘inactive epochs,’ as shown in Figure 2.

### EEG sources appear more synchronous that MEG sources

**Figure 3:**
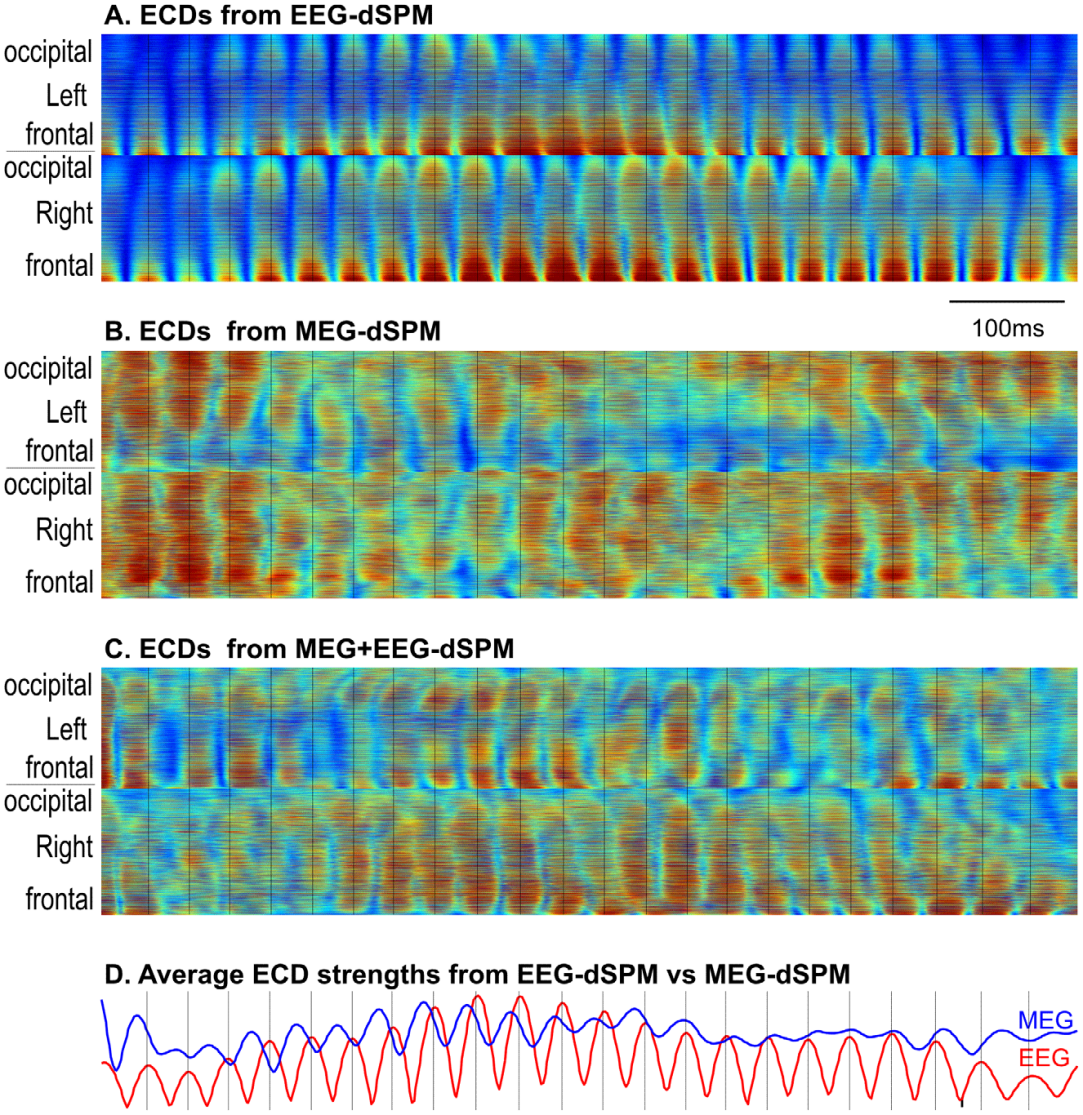
Source space electromagnetic profile of sleep spindles. Time-intensity plots of square root of dSPM F-statistics across all 6500 cortical locations as estimated for EEG-dSPM (panel A), MEG-dSPM (B) or both modalities (MEG+EEG-dSPM, panel C). Thin black vertical lines at the peaks of the EEG spindles are extended across all panels, showing that they are not aligned with the peaks of the MEG spindles. ECDs from EEG are highly synchronous across the cortex, whereas ECDs from MEG and M/EEG are rapidly shifting in phase, hemisphere, and the location within the hemisphere. ECD power at each time point averaged across all ECDs is shown in D. Coherence of these measures between EEG and MEG for this spindle was 0.7. For another example from another subject, see Figure S2.

Estimated dipole strengths in ∼6500 cortical locations during a sample spindle were color-coded and plotted as lines that were stacked vertically in Figure 3. ECDs derived from EEG-dSPM oscillated in synchrony, whereas those derived from MEG did not (Figure 3A,B; see also Figure S2). Note also that MEG-derived sources do not exhibit peak amplitude at the same moment as EEG-derived sources. Dipole estimates derived from the combined MEG and EEG measurements display an intermediate pattern (Figure 3C). This pattern of synchronous activity across the cortex when estimated from EEG, and asynchronous when estimated by MEG, was observed for every spindle analyzed.

### Dynamic sequence of cortical activity during spindling estimated with combined MEG/EEG

**Figure 4:**
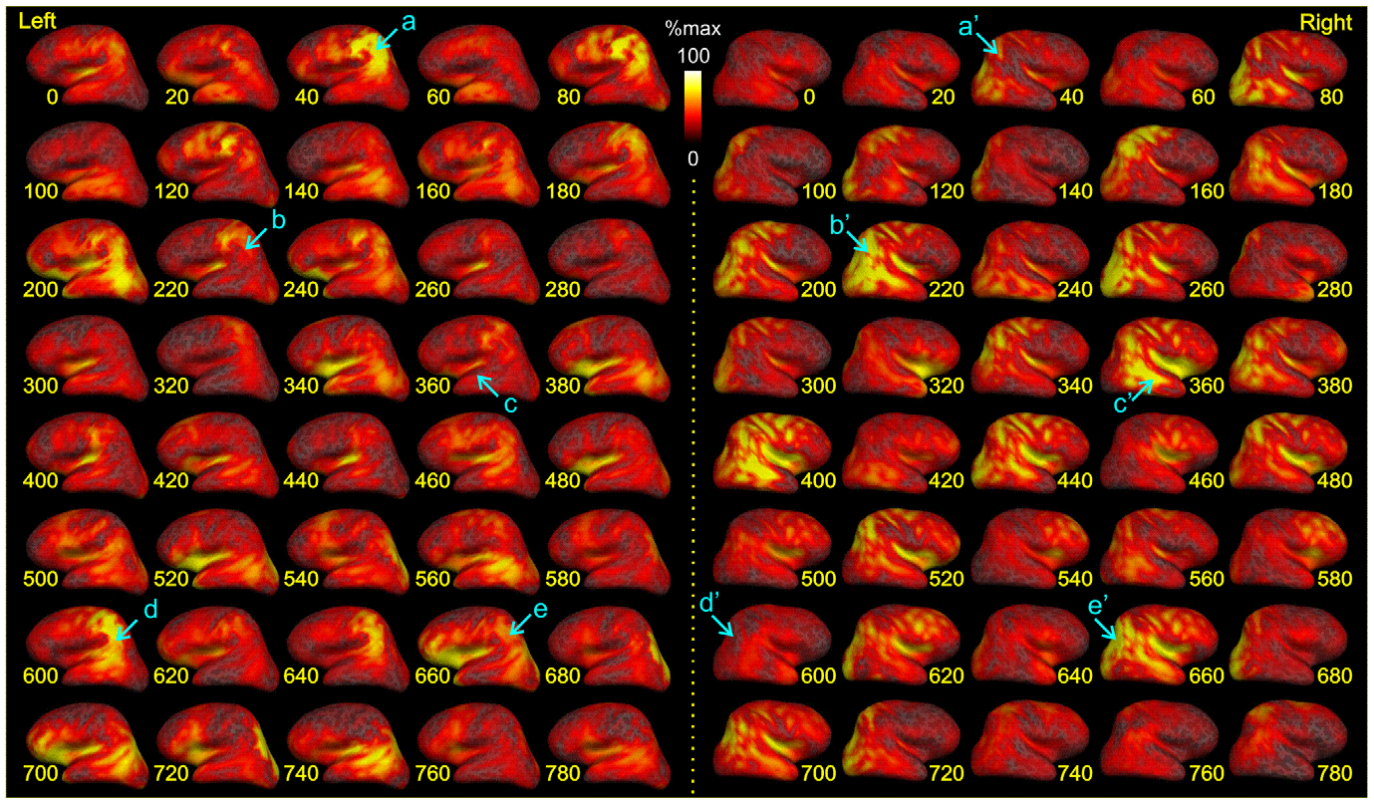
Dynamic spatiotemporal patterns of spindling. A combined MEG+EEG-dSPM solution is mapped on cortical surface throughout the duration of an example spindle. Each row shows 100 ms of left (on left) and right (on right) hemisphere activation maps with 20 ms delays between two consecutive snapshots. Note the variety of patterns for successive spindle waves within the same spindle discharge. For example, at 40 ms the spindle is estimated to arise mainly in the left parietal lobe (see blue arrow marked with an **a**), at 220 ms, right occipitotemporal (**b'**), and at 360 right temporal and perisylvian (**c'**) with relatively little contralateral activity (**a', b, c**). Later, at around 600 ms, maximal activity returns to left parietal (**d**) then around 660 shifts to the right hemisphere (**e'**) and so on. Estimated ECD strength is plotted on the subject's cortex after expansion to reveal sulcal as well as gyral cortex. Subj. 5.

Figure 4 portrays the estimated sequence of cortical activity during a sample spindle (from subject 5) calculated based on MEG and EEG combined. Examination of these snapshots (every 20 ms) suggests that spindles are not synchronized among different regions of the cortex, but rather the peaks in different areas are offset in time, all within a given spindle discharge. Furthermore, successive peaks of the spindle produce maximal activation in different locations. In particular, activities in the left and right hemispheres are not in synchrony with each other. At different times in the example spindle shown in Figure 4, maximal activity is seen in the left parietal, left orbital, left occipitotemporal, right occipital, right temporal, and right parietal. A comparable level of variability was found in each spindle in all subjects.

### Contrasting cortical dynamics during spindling estimated from EEG vs. MEG

**Figure 5:**
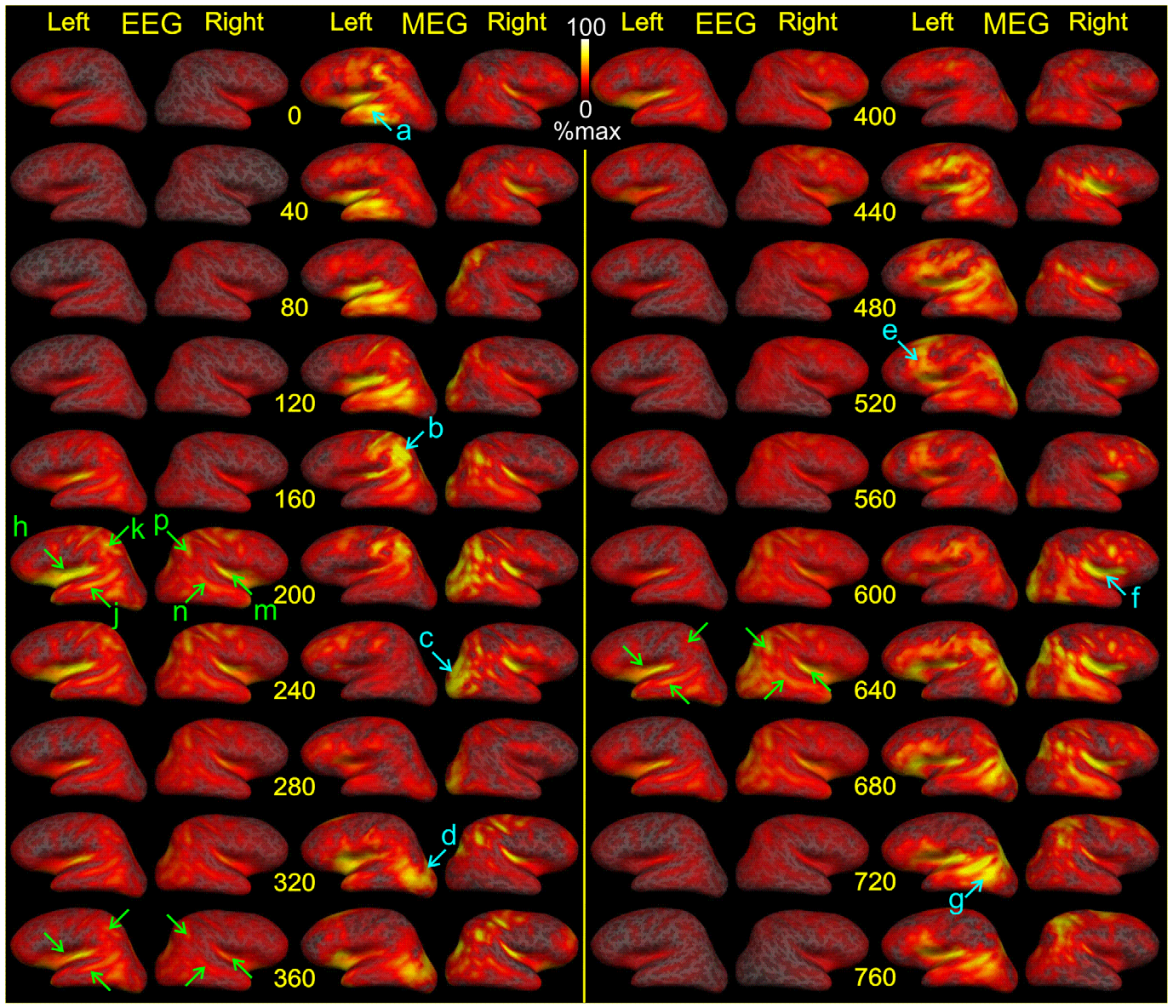
Dynamic spatiotemporal patterns of spindling. Contrasting dSPM solutions from MEG and EEG to simultaneous data, as mapped on cortical surface throughout the duration of a spindle. Time proceeds from top to bottom in each column, with successive snapshots separated by 40 ms. The left 4 columns show activity from 0 to 360 ms, and the right 4 columns from 400 to 760 ms, of the same spindle discharge. Note that activation peaks are not synchronous in MEG and EEG, nor are they in the same locations. MEG is highly variable across time, with successive peaks of activity (see blue arrows) in left temporal at 0 ms (**a**), left parietal at 160 (**b**), right occipital at 240 (**c**), left occipital at 320 (**d**), left frontal at 520 (**e**), right insula at 600 (**f**), and left occipitotemporal at 720 (**g**). In contrast, EEG-derived source localizations appear more bilaterally symmetrical and consistent over time. For example, at 200 ms, relatively high activation is estimated to the left and right insula (**h**, **m**), superior temporal sulcus (**j**, **n**), and parietal lobe (**k**, **p**). Very similar activation is seen at 360 and 640 ms (see green arrows). Estimated ECD strength is plotted on the subject's cortex after expansion to reveal sulcal (dark gray) as well as gyral (light gray) cortex.

Figure 5 contrasts the dSPM estimates derived from EEG and MEG in the same spindle, plotted on the subject's reconstructed cortex, after expansion to reveal sulcal as well as gyral cortex. Again, in the MEG-dSPM solution, maximal activity is estimated to different cortical lobes and hemispheres in different parts of the same spindle. In contrast, the anatomical pattern of activity estimated from EEG is relatively constant over time. Also note that the activity estimated from EEG versus MEG are maximal at different times during the spindle discharge.

### The overall average source distribution is dissimilar for MEG and EEG

**Figure 6:**
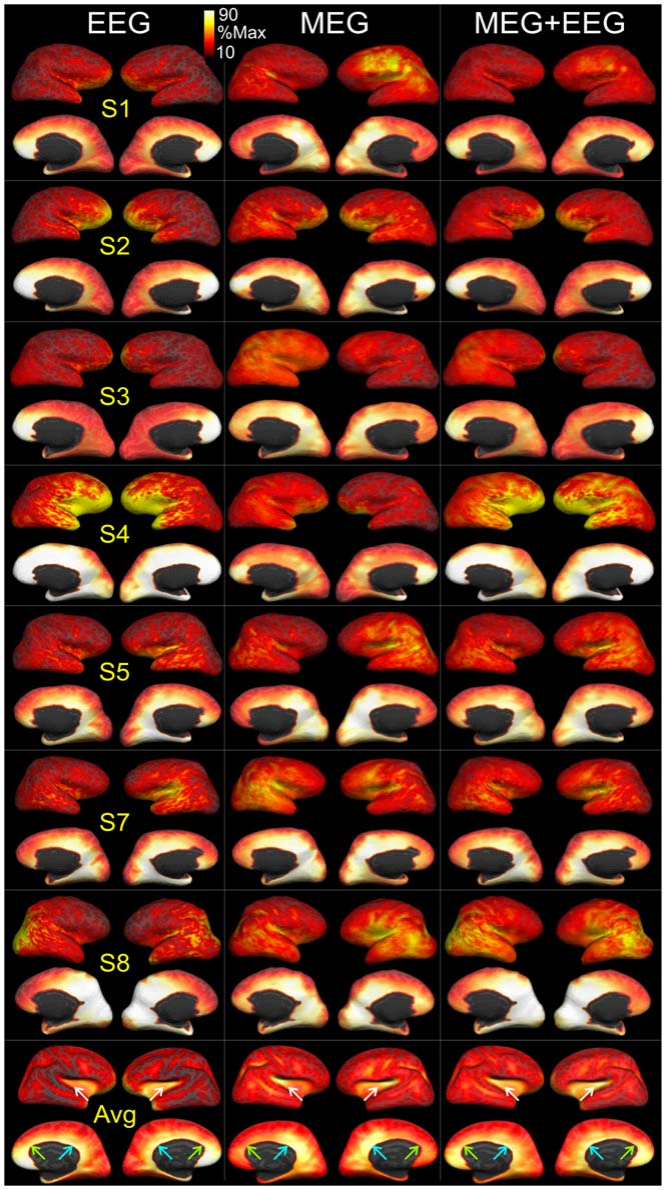
Maps of average estimated cortical activation during spindles. Activity was estimated with dSPM using EEG, MEG or combined EEG and MEG (MEG+EEG) data. For each spindle and modality, a map was made of the maximum activity during that spindle at each of ∼6500 cortical locations. These maps were then averaged across all spindles from that subject, normalized to the maximum value, and displayed below on the expanded cortical surface. The subject-specific cortical maps were then averaged together and plotted at the bottom of the figure. All subjects and modalities show maximal activity in medial cortex, varying across subjects between more anterior (green arrows, bottom row) and posterior (blue arrows) regions. Lateral activity is weaker and includes the insula (white arrows) and all lobes. The site of maximum lateral activity varies across subjects between frontal, parietal and temporal lobes.

Although the spindle thus appears to have a variable distribution over the cortical surface across time, it is possible that the total set of cortical areas active at some point in generation of a spindle burst is consistent across spindle bursts. In order to evaluate this possibility, we calculated the maximum of each cortical dipole's activation value during a given spindle burst. Next, by inter-spindle averaging of these maps of maximum activity, the overall cortex involved in spindle generation for an individual subject was estimated. This approach was applied to all three inverse solutions (calculated from EEG, MEG and both combined), and in Figure 6 they are mapped onto each individual's cortical surface. These images suggest that regardless of the measurement modality, this inverse method tended to place maximal activation in the deep midline areas, i.e., in the cingulate, subgenual, and parahippocampal areas. Secondary areas, variable across subjects and measurement modalities are also apparent. The similarity of these estimated source patterns was calculated as the correlation coefficient of the estimated average noise-normalized power across all cortical locations from EEG vs MEG. The average of this measure across the 7 subjects was 0.4± 0.13. If the estimated source localizations from MEG vs EEG were always the same for each subject, then the correlations would have been 1; if they are random, then the average would be zero. The observed low correlation indicates that largely dissimilar activation maps are inferred from EEG vs MEG.

### MEG and EEG sources are poorly correlated but moderately coherent

The inverse procedure estimates the timecourses for equivalent current dipole sources (ECDs) at about 6500 cortical locations. These estimates were made separately from MEG gradiometer and EEG referential recordings, and the between-modality correlation and coherence of such solution time courses were calculated at each cortical location. The results of these correlations were averaged across locations to obtain a single number for every spindle in each subject. This measure of the similarity of the source timecourses inferred from MEG versus EEG was very low, with a mean and std of 0.09±0.06. However, the similarity of these time courses, estimated from their coherence from 10–15 Hz at given locations between modalities, was much higher, with mean and std of 0.44±0.08 using the MVDR method and .54±0.16 using the Welch method. Since correlation but not coherence is sensitive to phase differences, this suggests that the modalities share a rhythmic pattern but are out of phase. Thus, at the source level, EEG- and MEG-derived solutions were poorly correlated but moderately coherent.

### Within-modality correlations indicate greater synchrony of EEG sources

The issue of synchrony of the inferred source activity across the entire cortical surface within each measurement modality was evaluated by calculating the correlation coefficient of the solution time-courses estimated at all possible cortical location pairs (∼21 million pairs for 6500 dipoles excluding self-pairs and repeats). The correlation coefficients were averaged to yield a single number for each spindle and the same process was repeated for all spindles in each subject. The average across subjects of this across-dipole correlation within the EEG modality was 0.64± 0.05. In contrast, MEG-dSPM had a much lower within modality correlation of 0.13± 0.01. These within modality correlation measures show that cortical sources estimated from EEG are more highly synchronous during spindles than those estimated from MEG.

## Discussion

The current study was motivated by our finding that signals recorded during spindles simultaneously by EEG and MEG sensors have strikingly different characteristics [23]. EEG was highly coherent across the scalp, with consistent topography across spindles. In contrast, the simultaneously recorded MEG was not synchronous, but varied strongly in amplitude and phase across locations and spindles. These differences were observed between the activity of EEG and MEG sensors during spindles, raising the question as to whether they would also be observed in the cortical activity inferred from the sensor activity. In the current paper we examined this question by first estimating the activity of cortical dipoles during sleep spindles using a distributed cortically-constrained source model (dSPM), separately from EEG and MEG, signals. We found that the location and timing of cortical activity inferred from EEG had a low correlation with that inferred from MEG. In agreement with sensor space measures, EEG-dSPM indicated a large-scale synchrony among different cortical sources. In contrast, MEG-dSPM applied to the same spindles estimated generation in shifting cortical locations, with simultaneously active generators that were largely independent of each other (and the EEG-dSPM) in frequency, phase and amplitude.

### Comparison to previously applied source localization methods

Our study appears to be the first that has directly compared source estimates to simultaneously recorded whole-head EEG vs MEG during sleep spindles. However, several studies have previously estimated sources to EEG or MEG spindles individually. Most often, a small number of ECDs were used to model the signals. Several workers found that four sources, placed in the deep parieto-central and fronto-central regions bilaterally, are adequate to explain most of the variation in spindles, including the tendency for frontal spindles to be slower [15], [17]. Urakami et al [15] specifically selected spindles based on their EEG frequency and topography. They fit a single ECD to ∼10–15% of the MEG channels, projected this activity out of the signal, then fit another ECD to another set of selected channels, and so forth until 80% of the signal was accounted for. The resulting dipoles clustered in the white matter midway between the lateral and medial surfaces of the cortex, deep in the Rolandic areas. Sources for both slower and faster spindles were found in both precentral and postcentral cortices, with a slight preference for slower spindles to be located in precentral areas and faster in postcentral. Manshanden et al. [17] similarly found that the ECDs, which best modeled MEG signals during spindles, were clustered in the white matter underlying centro-parietal, parietal and posterior frontal cortices. Shih et al. [19] also modeled MEG spindles using small numbers of ECDs, located in all lobes across different spindles, but their subjects were sedated, and this may tend to cause spindles to be less synchronous [6]. Using Synthetic Aperture Magnetometry (SAM), Ishii et al. [16] also located the sources of MEG spindles mainly in the white matter underlying frontal and parietal cortices. Using another distributed solution (ICA followed by MR-FOCUSS), Gumenyuk et al [18] estimated maximal source activity to frontal, temporal and parietal lobes. Most commonly, maximal activity was in Rolandic cortex, with overlapping sources for faster and slower spindle components. In a study estimating sources from EEG using LORETA (low resolution brain electromagnetic tomography), Anderer et al. [42] localized activity to the medial parietal and frontal cortices, with more frontal areas associated with lower spindle frequencies.

The electromagnetic inverse problem is ill-posed; arriving at a solution requires a priori assumptions whose validity is generally unknown [43]. Despite their contrasting assumptions, the above studies generally yielded consistent results, estimating maximal activity during spindles to the white matter underlying parietal and frontal cortices. Since distributed sources generally result in equivalent dipoles that are deep to the generating surface [44], the previous results are consistent with distributed generators in parietal and frontal cortices. To a limited extent, direct intracranial measures have provided some validation of these conclusions. Several studies have recorded sleep spindles over lateral and medial prefrontal [45], [46], medial temporal [46], [47], and parietal cortices [48].

When averaged across all time points, spindles and subjects our results resemble previous results, being maximal in medial parietal, central and frontal areas (Figure 6). However, since sources are constrained by dSPM to the individual subject's cortical surface, our results are unlike previous findings in that the activity is not localized to the white matter. Furthermore, our focus here is not on the location of the spindle sources but on a comparison of the spatiotemporal dynamics within individual spindles of the EEG vs MEG inverse estimates, a question that has not been examined previously.

### Contrasting characteristics of MEG and EEG in source space

We used several methods to examine the synchrony of estimated source activity during the spindle between different cortical locations. The stacked amplitude plots from EEG-dSPM (Figure 3A) showed synchronous activity in the ∼6500 ECDs tiling the cortical surface, while those estimated from MEG-dSPM (Figure 3B) indicate peaks at different times in different locations. Not only the amplitudes but also the frequency and phase vary across locations for MEG-derived ECDs, but not EEG-derived ECDs. Similar differences are observed when the estimated ECD amplitudes are plotted on the cortical surface reconstructed from the MRI of each individual, as sequential topographical snapshots (Figure 5). Although the power of cortical activation estimated from EEG varies across the course of a spindle, its pattern remains relatively constant. In contrast, the maximal activity estimated from MEG jumps rapidly between cortical areas and hemispheres, all within the same spindle discharge. These contrasting characteristics were observed in all 85 spindles sampled from the 7 subjects in the study. We quantified the degree of synchrony as the average correlation coefficient between the estimated activity time courses in different cortical locations. The average correlation across spindles and subjects of EEG-derived ECDs was 0.64, and for MEG-derived ECDs was 0.13. Thus, the stacked amplitude plots, sequential topographical snapshots, and average correlation coefficients all demonstrate that the cortical sources estimated from EEG are highly synchronous during spindles whereas those estimated from MEG are much less synchronous.

The fact that sources estimated from EEG vs MEG have very different characteristics implies that they are poorly correlated with each other. Indeed, the peaks of activation as observed with EEG vs MEG occurred at different times as indicated by stacked amplitude plots (Figure 3A vs 3B ), or cortical topography snapshots (Figure 5). When the time courses of all cortical dipoles are added together, the peaks of activation for EEG and MEG are seen to not only misalign, but to shift rapidly in phase and relative amplitude (Figure 3C). We quantified the degree of synchrony in EEG vs MEG solutions as the correlation coefficient between the activities estimated at the same cortical location with the different modalities. This correlation, averaged across cortical locations, spindles and subjects was very low (0.09), despite the fact that these recordings were made simultaneously and source estimates were obtained using identical inverse methods.

### Why are MEG and EEG sources different?

We conclude that in source space, using the dSPM method, EEG and MEG during spindles have highly contrasting characteristics during sleep spindles. There are two possible explanations for these findings. One is that our inverse estimates are correct, and EEG vs MEG sleep spindles are generated by different cortical sources with different characteristics. The second is that our inverse estimates are incorrect, mis-estimating either the EEG or MEG sources, or both, resulting in the incorrect conclusion that their sources are asynchronous and distinct.

The ultimate sources of both EEG and MEG signals are active transmembrane currents, balanced by passive transmembrane return currents. Intracellular currents linking active and passive transmembrane currents generate MEG, and extracellular currents generate EEG. The fact that EEG and MEG are thus generated by different limbs of the same circuit would lead one to assume that their sources should generally be estimated to the same locations. However, recent studies have suggested that differences could arise because, in fact, for distributed sources such as the sleep spindle, most of the electrical or magnetic signal that is generated in the cortex never arrives at the sensor, and that which does arrive at the sensor is different for EEG vs MEG. Simulations with actual cortical architectures show that co-activation of just 1% of the cortical dipoles results in cancellation of over 90% of their signal due to cortical folding [49]. For example, co-activation of dipoles lying on opposite sides of a sulcus may result in near-total cancellation [50]. In addition, MEG is relatively insensitive to radial dipoles, whereas EEG is sensitive to dipoles that are either radial or tangential with respect to the skull [51]. It is essential to recognize that inverse estimators attempt to localize only the origins of signals that reach the sensors, not all of the dipolar activity in the cortex. Thus, it is entirely possible that our inverse estimates are correct and the EEG and MEG during spindles do arise from different sources.

A second argument suggesting that our inverse estimates may be in error in ascribing different cortical generators to the EEG versus MEG is that we could successfully estimate cortical source distributions that seemed to account for both the MEG and EEG data (Figures 4 and 6). However, our simultaneous inverse solution attempted to fit the spatial patterns of MEG and EEG but not their relative amplitudes, because arriving at the correct scaling factor requires data from a known single tangential dipole such as the initial response to median nerve stimulation, which was not available in this study [33]. A consideration of the absolute amplitudes of MEG and EEG spindles suggests that this may be critical for an accurate simultaneous solution. On the one hand, the ratio of EEG amplitude recorded at the cortical surface to that recorded at the scalp during spindles is about 2∶1 [46], [48], [52], consistent with an extremely widespread generator [53]. Conversely, a focal source generating a MEG spindle of the observed size would produce an EEG spindle about 50x smaller than that actually observed [33], [54]. Thus, it is entirely possible that a simultaneous dSPM solution which estimates cortical sources reproducing the relative amplitudes of the MEG and EEG signals as well as their spatial distribution may estimate both a distributed diffuse synchronous generator which contributes significantly to EEG but not MEG, and multiple asynchronous relatively focal generators which contribute significantly to MEG but not EEG. In addition to fitting the absolute amplitudes of the MEG and EEG signals (and not only their topographical patterns), future modeling studies should include the CSF layer under the skull and the anisotropy of cerebral white matter, which affect the size and distribution of the EEG signal to cortical dipoles [55], [56].

Previous reports have shown that some spindles are recorded by MEG but not EEG, and vice versa [17], [20], [21], [22], and that intracranially recorded spindles often have no clear or consistent relationship to the spindles recorded simultaneously at the scalp [46], [47], [48], [52]. These observations would also be consistent with the view that MEG and EEG are recording from different brain systems during spindles.

Indeed, studies in animals have demonstrated that, although cortical spindles can be widely synchronous, they can also be restricted to small thalamo-cortical modules, oscillating in multiple areas, with largely independent durations, onsets, frequencies and phase [4]. These distributed and focal spindles were interpreted in terms of the ‘recruiting’ and ‘augmenting’ responses that characterize thalamo-cortical projections from the ‘non-specific intralaminar’ and ‘specific projection’ nuclei respectively [5]. This distinction has evolved into a distinction between the ‘matrix’ and ‘core’ thalamo-cortical systems [57]. Thalamo-cortical cells in the matrix system project widely, even to multiple cortical areas, terminating with small boutons in layer I; in contrast, thalamo-cortical cells in the matrix system may project to a single column, terminating with large boutons in layer IV [58]. Matrix cells are found in all thalamic nuclei, but predominate in intralaminar and other nonspecific nuclei; core cells are concentrated in the specific sensory relay nuclei.

Thus, classical studies of spindle discharges in cats demonstrated both distributed and focal spindles which apparently reflect activation of the matrix and core thalamo-cortical systems, respectively. The differences between EEG versus MEG spindles may arise from their biophysically-determined differential sensitivity to these different thalamocortical systems, with EEG more sensitive to diffuse activation via the matrix system, and MEG to focal activation via the core system. The current study demonstrates that these contrasting characteristics are clearly seen in the cortical sources of MEG and EEG spindles, as estimated with dSPM.

## Supporting Information

Figure S1 Example spindles. Selected spindles in all EEG and MEG channels are highlighted in yellow.
(7.02 MB TIF)

Figure S2 Source space electromagnetic profile of sleep spindles. Time-intensity plots of the square root of dSPM F-statistics across all 6500 cortical locations as estimated for EEG-dSPM (A), MEG-dSPM (B) or combined solution (MEG+EEG-dSPM) as shown in C. ECDs from EEG are highly synchronous across the cortex, whereas ECDs from MEG and M/EEG are rapidly shifting in phase, hemisphere, and the location within the hemisphere. ECD power at each time point averaged across all ECDs is shown in panel D.
(1.05 MB TIF)
